# An anatomically shaped medial meniscus prosthesis is able to partially restore the contact mechanics of the meniscectomized knee joint

**DOI:** 10.1186/s40634-022-00531-6

**Published:** 2022-09-07

**Authors:** Branco S. van Minnen, Albert J. van der Veen, Sebastiaan A. W. van de Groes, Nico J. J. Verdonschot, Tony G. van Tienen

**Affiliations:** 1 Orthopaedic Research Lab, Radboud University Medical Centre, P.O. Box 9101, 6500 HB Nijmegen, The Netherlands; 2 ATRO Medical B.V., Liessentstraat 9A, 5405 AH Uden, The Netherlands; 3 Department of Orthopaedics, Radboud University Medical Centre, P.O. Box 9101, 6500 HB Nijmegen, The Netherlands; 4 Department of Biomechanical Engineering, University of Twente, Enschede, The Netherlands

## Abstract

**Purpose:**

The aim of this study was to determine whether a flexible medial meniscus prosthesis is more capable of sharing loads with the direct tibiofemoral cartilage contact than the stiffer first-generation prosthesis. Additionally, the effect of the prosthesis on the tibial pressure distribution after total meniscectomy was investigated.

**Methods:**

In an artificial knee joint, the relative amounts of load transferred through both meniscus prostheses and the direct tibiofemoral contact were assessed with pressure-sensitive sensors.

Additionally, six cadaveric knee joints were loaded in a physiological environment. Tibial contact pressures were measured with an intact native meniscus, after total meniscectomy and after implantation of the second-generation meniscus prosthesis.

**Results:**

Whereas the first generation of the meniscus prosthesis transferred virtually all the load from femur to tibia, the second-generation prosthesis allowed for load sharing with the direct tibiofemoral contact.

No differences in load sharing were found between the native meniscus and the second-generation meniscus prosthesis. The prosthesis decreased peak and mean pressures on the medial tibial cartilage compared to meniscectomy. No significant differences in pressure were found between the native meniscus and the meniscus prosthesis.

**Conclusions:**

The second-generation meniscus prosthesis presented in this study can share loads with the direct tibiofemoral contact, a characteristic that the first-generation prosthesis did not have.

The flexible meniscus prosthesis significantly reduces the contact pressures on the medial tibial plateau after total meniscectomy. Although the biomechanical performance of the native meniscus could not be reproduced completely, the meniscus prosthesis may have the potential to relieve post-meniscectomy pain symptoms.

## Introduction

The most common knee injuries are meniscal tears [15], which can be caused either by acute trauma or by degenerative processes [6]. This results in over 500,000 partial or total meniscectomies in the U.S. [1, 16] and Europe [17] every year. However, the removal of meniscal tissue, is a strong predictor of knee osteoarthritis and pain [4, 5, 18]. Tibiofemoral contact pressures are known to increase after (sub-)total meniscectomy [2, 14, 15, 21, 31], which may be one of the causes of the pain experienced by patients after undergoing meniscectomy. Meniscal allograft transplantation (MAT) decreases tibiofemoral contact pressures [11, 27] and provides an off-the-shelf possibility to relieve pain and delay the onset of knee osteoarthritis [23]. However, the availability of size-matched meniscal allografts is limited and the long-term clinical effects may be affected by extrusion [13, 23] or shrinkage of the allograft [12, 19].

An anatomically shaped, polycarbonate urethane (PCU) meniscus prosthesis was developed, based on the average meniscus geometry obtained from MRI scans of 35 healthy subjects [26]. It was designed to restore the biomechanical function of the native medial meniscus without the disadvantages of MAT. Pre-clinical studies showed that prototypes of the prosthesis were able to reduce the tibiofemoral pressures relative to the meniscectomized knee joint, to similar levels as an allograft [10, 27]. None of these studies, however, involved testing in a wet environment at body temperature, i.e. in physiological conditions.

**Fig. 1:**
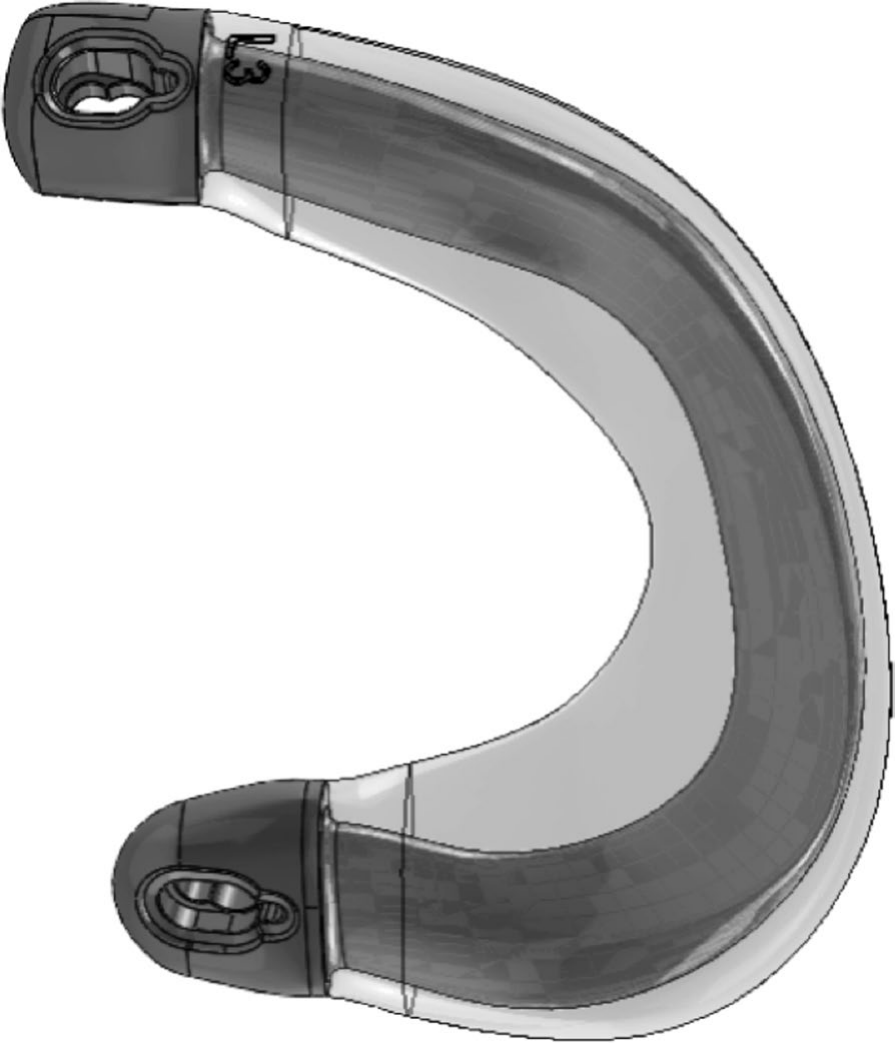
First-generation meniscus prosthesis, with a reinforcing core and soft outer layer

The first generation of the prosthesis was a two-component implant, with a stiff reinforcing core to limit extrusion and a soft cartilage-contacting layer (Fig. 1). Two titanium fixation screws were developed to secure the prosthesis to the anatomical attachment sites of the meniscal horns on the tibial plateau. This resulted in a relatively rigid prosthesis, which requires a perfect fit and position due to the lack of geometrical adaptability, to conform with the femoral surface geometry which changes during flexion and extension. This configuration was evaluated during a first-in-human clinical investigation in five patients, who had previously undergone meniscectomy [24]. The high in-plane stiffness of the prosthesis, in combination with the rigid fixation technique by means of screws, resulted in a deficit of knee flexion and extension and persistent pain in all patients. Four out of five prostheses were explanted, of which three were broken. It was concluded that, due to the rigidity of the prosthesis and its fixation, the compressive loads were transferred entirely through the prosthesis and not through the direct tibiofemoral contact [24]. This lack of load sharing increased the compressive and circumferential stresses in the prosthesis and ultimately resulted in structural failure of the prosthesis. Furthermore, it was expected that in the relatively high temperature inside the human body, the water uptake of the PCU would result in a more flexible prosthesis [7]. Less adaptation of the prosthesis than expected may have contributed to the failure mechanism described in the first-in-human clinical investigation. Earlier studies investigating the effect of water uptake and temperature on the prosthesis are lacking.

**Fig. 2:**
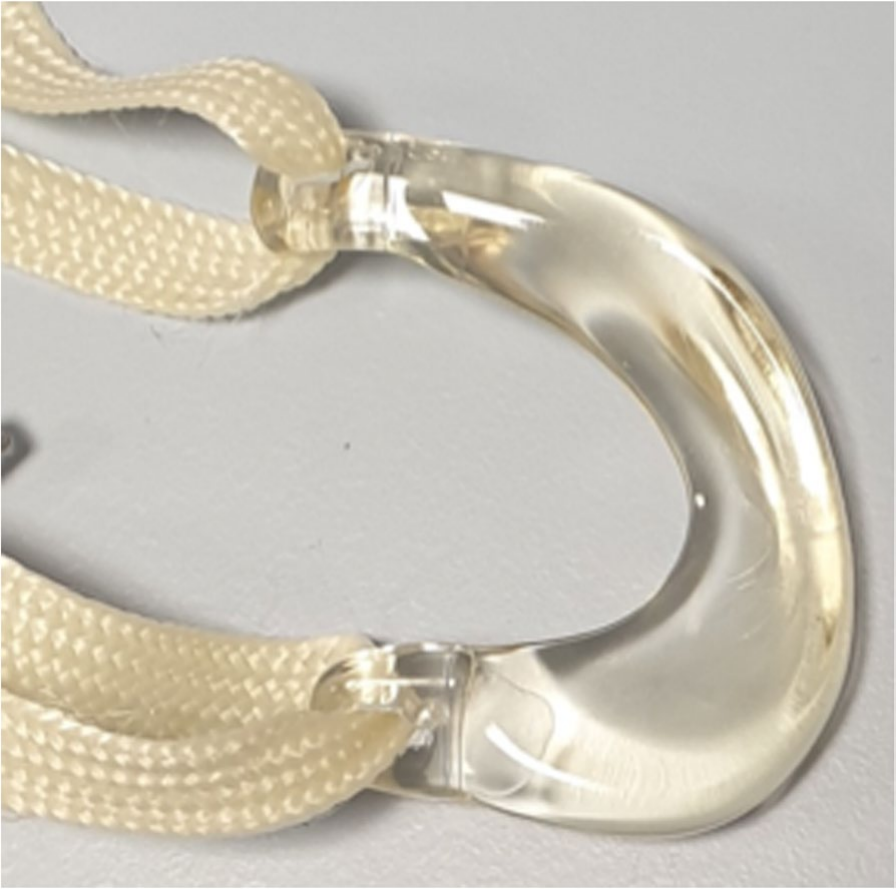
Second-generation meniscus prosthesis, with strong horns, a flexible body and an anchoring tape

To overcome the issues that were identified during the first clinical investigation, the meniscus prosthesis and its fixation technique were adapted, resulting in a more flexible and adaptable device (Fig. 2). The first objective of this study was to assess the load sharing capabilities of the flexible second-generation prosthesis in an artificial knee joint. It was hypothesized that a more flexible prosthesis would allow for more load sharing with the direct tibiofemoral cartilage contact. In the same test, the effects of temperature and water uptake were investigated to obtain a better understanding of the material properties in different conditions. A more flexible prosthesis could potentially lead to excessive extrusion from the joint and, with that, possible loss of function. Therefore, the second objective of this study was to investigate the functional biomechanical performance of the second-generation medial meniscus prosthesis system in different cadaveric knee joints, measured by contact pressures on the tibial plateau. The prosthesis is expected to improve the contact mechanics compared to a total medial meniscectomy, in a similar way to the allograft.

## Materials and methods

**Fig. 3:**
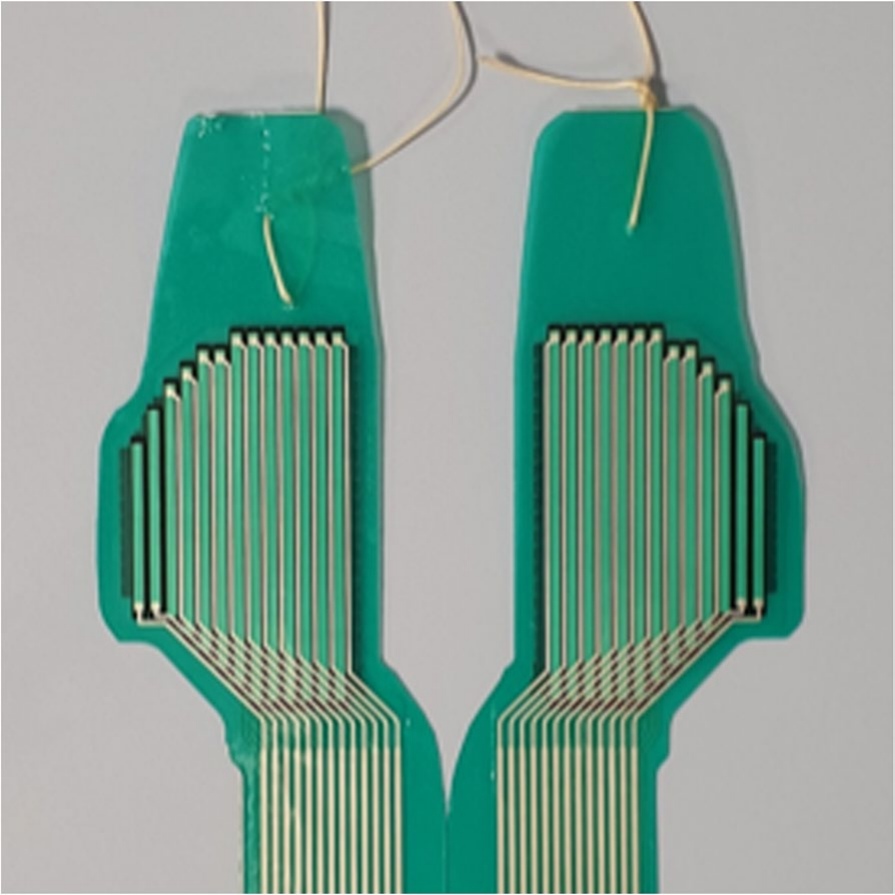
Tekscan 4011 pressure-sensitive sensor

**Fig. 4:**
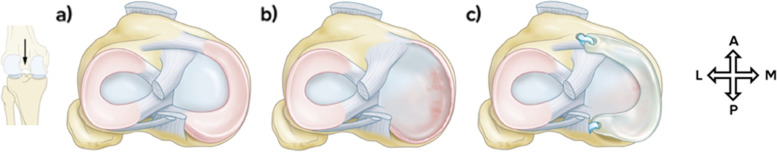
Schematic representation of the different situations tested in the cadaveric knee joints. **a** Native menisci. **b** Total medial meniscectomy. **c** Second-generation medial meniscus prosthesis. The arrows indicate the anterior (A), posterior (P), lateral (L) and medial (M) sides of the tibial plateau

**Fig. 5:**
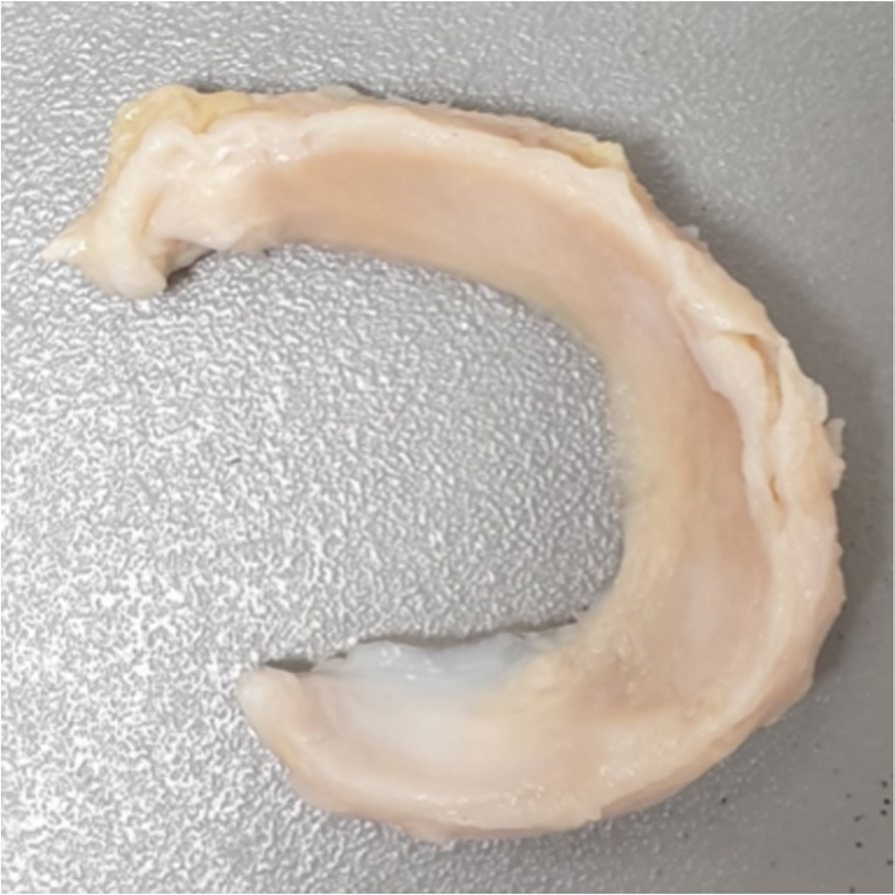
Native medial meniscus, excised from a cadaveric knee joint

This study consisted of two experiments in different test setups: In an artificial knee joint, the load sharing capabilities of the second-generation meniscus prosthesis (Fig. 2) were compared to the first generation (Fig. 1). The effects of water uptake of the meniscus prosthesis and environmental temperature were investigated as well. In the second test, axial loads were applied to cadaveric knee joints under physiological knee conditions. Contact mechanics on the medial and lateral tibial plateau were evaluated with pressure-sensitive sensors (Tekscan, South Boston, MA, USA, Fig. 3), in three situations (Fig. 4): With both intact native menisci (Fig. 5), after total medial meniscectomy and with the second-generation artificial medial meniscus prosthesis implanted.

### Meniscus prostheses

The rigid first generation of the medial meniscus prosthesis consists of a stiff core of Bionate® 75D polycarbonate urethane (PCU, DSM Biomedical, Berkeley, CA, USA), which extends into two meniscal horns of the same material. This reinforcing core is covered by a soft, cartilage-contacting outer layer of Bionate® II 80A PCU (Fig. 1). The horns of the meniscus prosthesis are fixed to the tibial plateau by using two dedicated fixation screws, made of a titanium alloy (Ti-6Al-7Nb).

For the design of the second-generation meniscus prosthesis (Fig. 2), two changes were implemented: Firstly, the stiff reinforcing core was removed, resulting in a single-component, soft and flexible meniscus body made of Bionate® II 80A PCU. The attachment horns of the prosthesis remained the same and are made of the stronger and stiffer Bionate® 75D PCU. Secondly, the titanium fixation screws were replaced by a polyethylene terephthalate (PET) anchoring tape. This tape is currently used clinically for fixation of ACL grafts and is secured on the anterior side of the tibia with polyether ether ketone (PEEK) anchoring screws.

All evaluated meniscus prostheses were manufactured by injection moulding of PCU, followed by EtO (ethylene oxide) sterilization. Since the approximately 1% water uptake is expected to strongly affect the mechanical properties of both materials, the wet-tested prostheses were pre-soaked for at least three weeks prior to testing [7]. Only average-sized (size 3) meniscus prostheses were used during this study, to match the selected cadaveric specimens.

### Test setup—Artificial knee joint

**Fig. 6:**
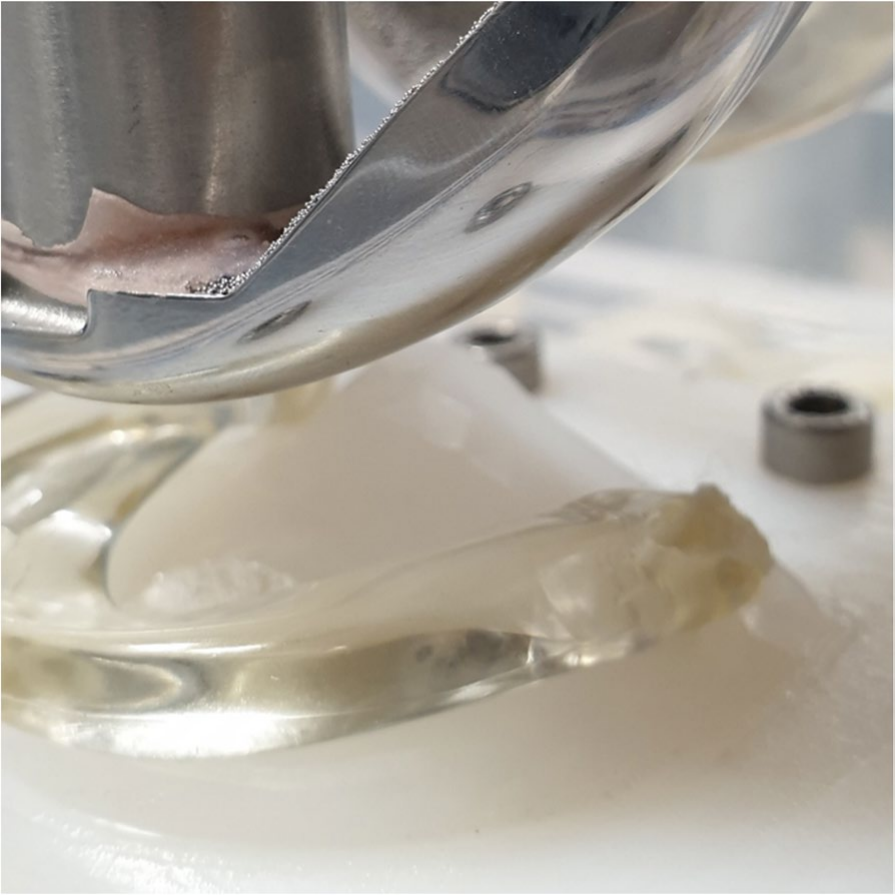
Second-generation meniscus prosthesis in the artificial knee joint

**Fig. 7:**
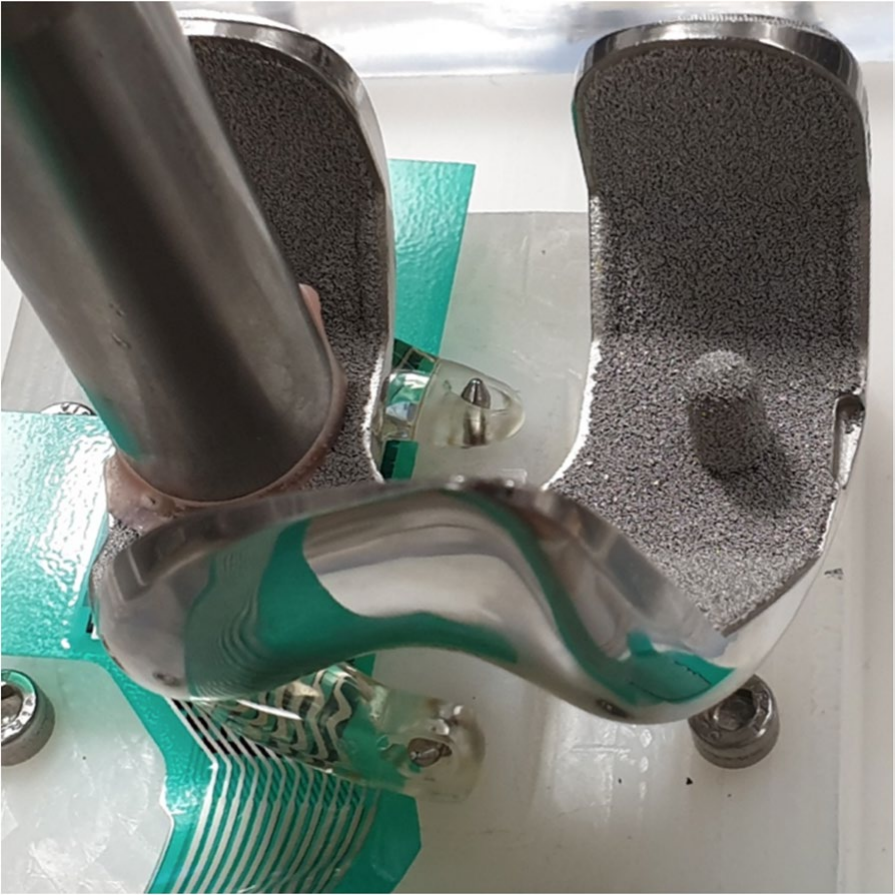
First-generation meniscus prosthesis and sensor in the artificial knee joint

As no test setup for assessing the load sharing between the meniscus prosthesis and the direct tibiofemoral contact has been described, an artificial setup was developed (Fig. 6). The medial tibial plateau of an average-sized knee joint was segmented from a CT-scan and machined from ultra-high molecular weight polyethylene (UHMWPE). The bottom side of the plateau was hollow, to reproduce the deformation that occurs in the tibial and femoral cartilage of the human knee joint [8]. The different meniscus prostheses could be placed on the tibial plateau by use of two pre-drilled fixation holes, suitable for both the screw and tape fixation techniques. The femoral part of the test setup consisted of an average-sized femur component of a total knee replacement, made of a cobalt chrome alloy (CoCr). Axial loads could be applied to the femur condyle by a hydraulic testing rig (MTS Systems, Eden Prairie, MN, USA). The test setup (Fig. 7) was placed inside a temperature-controlled water bath. The measurements were performed both dry at room temperature and in a water bath of 37 ± 2 °C.

### Test setup—Cadaveric knee joints

**Fig. 8:**
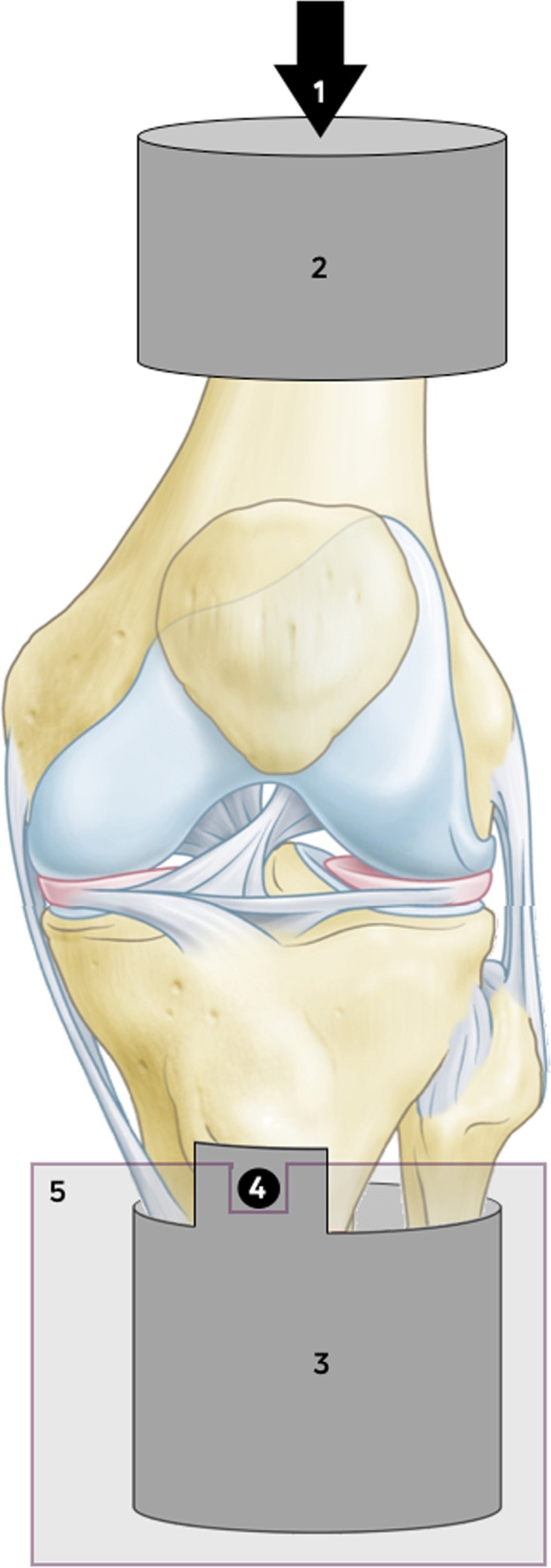
Schematic representation of the cadaveric knee test setup: 1 Axial load applied by the testing rig. 2 Femoral bone cup, restricted in all directions except for proximodistal translation. 3 Tibial bone cup and 4 Steel rod of ø8mm, allowing small anteroposterior translations and 14° of varus and valgus rotation. 5 Support for the steel rod, restricted in all directions

The femoral and tibial shafts of all specimens were shortened to approximately 12 and 10 cm respectively, to avoid any unnecessary effects of bending. Soft tissue was removed from the ends of the bones to enable potting of the bones with polymethyl methacrylate (PMMA) bone cement in stainless steel cups, which could be mounted in the test setup (Fig. 8). The setup was positioned in a water bath with a temperature of 37° ± 2 °C, to try to mimic the physiological conditions inside the knee joint [9]. It was decided to perform all tests in full extension, for better reproducibility of the alignment of the joints during testing and to maintain knee stability. The tibial cup was mounted in a dedicated support, using a steel rod oriented in the anteroposterior direction to make varus/valgus rotation of the knee joint possible. The axis of rotation was located approximately 5 mm medially of the centre, to allow for the unequal physiological load distribution between the medial and lateral compartment [9, 20]. Anteroposterior and mediolateral translations were not possible in this setup. Axial loads could be applied to the femoral bone cup by the MTS hydraulic testing rig.

### Specimens

After obtaining ethical approval from the Anatomy Department, fresh frozen human cadaveric knee joints were obtained from the Radboud University Medical Centre (Nijmegen, The Netherlands). Anteroposterior and mediolateral radiographs were used to select one right and five left average-sized knee joints, to match the size of the pressure-sensitive sensors and the available meniscus prostheses. Specimens were excluded if they showed significant signs of osteoarthritis, such as osteophytes and joint space narrowing. The absence of osteoarthritis was confirmed during the surgical procedure.

### Cadaveric surgery

All surgical activities were performed by an experienced orthopaedic knee surgeon (TvT). Firstly, all excessive skin, fat and muscle tissue was removed from the cadaveric knee joints. To improve visibility and to prevent folding of the pressure-sensitive sensor during insertion, the patella and the anterior and posterior parts of the joint capsule were removed. Care was taken to leave the collateral and cruciate ligaments intact, to maintain stability and physiological alignment of the knee joint. The meniscal horn attachments and the meniscocapsular and meniscotibial ligaments were left intact, while the periphery of the native medial and lateral menisci was detached from the tibial plateau. In this way, the pressure-sensitive sensor could be inserted underneath the menisci without folding. Detachment of the circumferential fixation is not expected to affect kinematics of the native meniscus [28], which is therefore expected to maintain its functional performance.

After the native condition was tested, the medial meniscus was completely removed by cutting both horn attachments and the attachment to the remainder of the joint capsule. After insertion of the pressure-sensitive sensor, the meniscectomy measurements were performed.

**Fig. 9:**
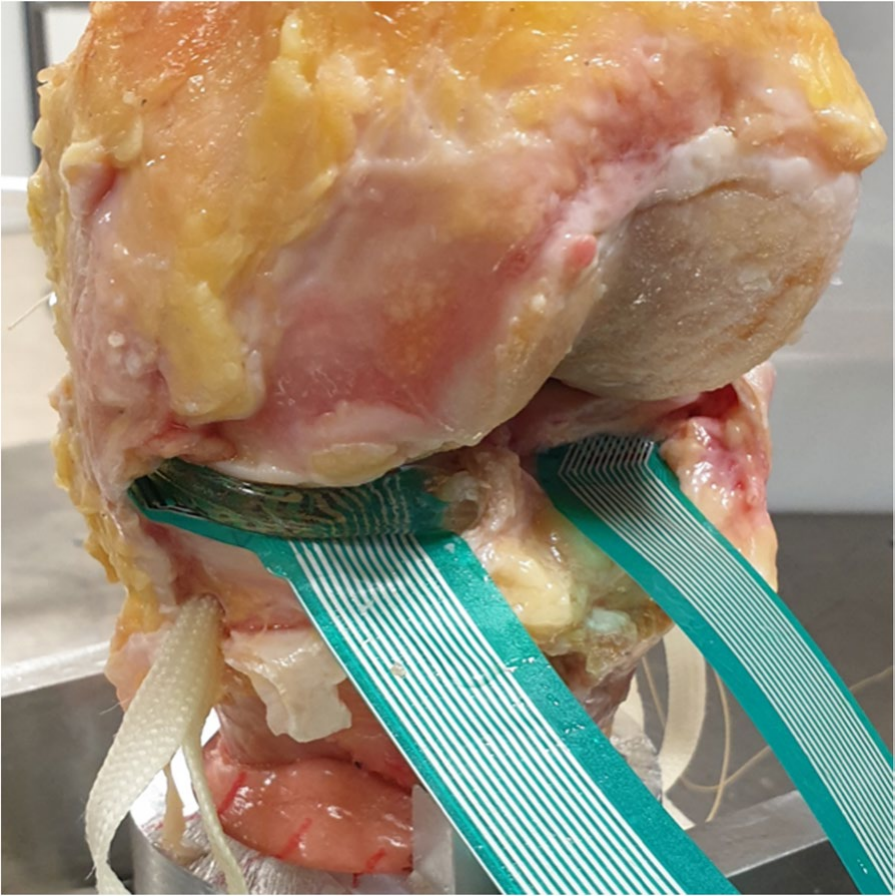
Second-generation meniscus prosthesis and sensor in a cadaveric knee joint

Subsequently, the second-generation medial meniscus prosthesis was implanted. Firstly, both anchoring tapes were led through the fixation holes of the prosthesis horns. A bone tunnel was drilled from the posterior horn attachment of the native meniscus to the anteromedial aspect of the tibia. By pulling the anchoring tape, the prosthesis was inserted into the knee joint, and the posterior horn was positioned above the tunnel. The optimal location of the anterior drill hole was determined with the knee in extension, thus preventing impingement or extension deficit. After drilling the anterior bone tunnel to the anterolateral aspect of the tibia, both anchoring tapes were tensioned and fixed in the tunnel by a PEEK anchoring screw. The pressure-sensitive sensor was positioned under the meniscus prosthesis before performing the final measurements, as shown in Fig. 9.

### Loading protocol – Cadaveric knee joints

Before each series of measurements in the cadaveric knee joints, three pre-conditioning cycles of 1000 N were applied to allow the knee joint to find its natural alignment. Subsequently, the measurements were performed at the end of 120 s of constant axial loading of 500 and 1000 N respectively. Although 1000 N is below physiological loads, the load was not increased any further, to prevent breakage of the cadaveric bones, which has occurred in pilot experiments. In between load cycles, the joint was always unloaded for at least 30 s.

### Loading protocol – Artificial knee joint

Approximately 70% of the physiological load is transferred through the medial joint compartment [20, 32]. Therefore, axial loads of 350 and 700 N were applied in the artificial knee joint, which only consist of a medial tibial plateau, to allow comparison with the 500 and 1000 N applied in the cadaveric tests.

### Pressure measurements

For this study, piezoelectric pressure mapping sensors of type 4011 (Fig. 3) were used for the measurements in both the artificial and the cadaveric knee joints. Prior to use, the sensors were pre-conditioned and calibrated by applying five different pressures between 0 and 7 MPa. In between the measurements of the different meniscal conditions, additional calibration measurements were performed to evaluate and correct for any possible loss of sensor sensitivity.

From the obtained pressure maps, the peak pressure, mean pressure and contact area on the medial tibial plateau were determined. Furthermore, the load sharing ratio between the medial meniscus or medial meniscus prosthesis and the direct tibiofemoral cartilage contact, i.e. the percentage of the medial compartment load that is transferred through the meniscus, was estimated from the pressure maps. Finally, the pressures on the lateral tibial plateau of the cadaveric knee joints were determined, to assess a potential shift in the load distribution from one joint compartment to another.

### Statistical analysis

Based on a previous study [27] and preliminary results from pilot testing, a sample size calculation was performed to determine the number of cadaveric specimens required. Using a power of 80% and a significance level of 0.05, six knees are required to detect contact pressure differences of 1.0 MPa, assuming a standard deviation of 0.5 MPa.

Linear mixed models were used to study the effect of meniscal condition (i.e. native meniscus, meniscectomy and prosthesis) of the medial knee compartment on contact mechanics. Medial peak pressure, mean pressure and contact area were analysed separately. In addition, the percentage of the load transferred through the meniscus or prosthesis and directly through the cartilage was analysed. Finally, the lateral mean pressure was analysed. The models included specimen as a random factor and all other variables that applied to the outcome measure under analysis (i.e. meniscal condition and axial load) as fixed factors. A random intercept was included to account for the specimen’s individual response to each experimental condition. Interaction terms between the fixed factors were also evaluated. Pairwise comparisons between the different levels of the fixed variables were performed by Tukey’s tests that were Bonferroni-corrected to account for multiple comparisons. 95% confidence intervals were determined and P-values below 0.05 were considered statistically significant. Statistical analyses were performed using R (version 4.1.2; R Foundation for Statistical Computing, Vienna, Austria).

## Results

### Test in artificial knee joint

No excessive extrusion was observed visually for any of the prostheses during the test, meaning that most of the prosthesis surface maintains in contact with the tibial plateau and femur condyle.

The results listed in Table 1 show that the first-generation meniscus prosthesis, with the rigid reinforcing core, transfers virtually all the load from artificial femur to artificial tibia. This is also the case when the flexible second-generation prosthesis is tested dry at room temperature. When this prosthesis is pre-soaked and tested under more physiological conditions, the load distribution shifts towards the direct tibiofemoral contact, especially when increasing the applied axial load on the femur. As this does not occur with the first-generation meniscus prosthesis, the difference in the relative amount of load transferred through the meniscus prosthesis is large, with 99% for the first generation and 36% for the second generation respectively at 700 N.

**Table 1:** Load sharing^a^ in the artificial knee setup

Force	Prosthesis	Dry, RT^b^	Wet, 37 °C
350 N	First generation	100%	100%
	Second generation	97%	92%
700 N	First generation	100%	99%
	Second generation	94%	36%

^a^ Percentage of load transferred through the prosthesis

^b^ Room temperature

### Test in cadaveric knee joints

In general, the size of the meniscus prosthesis matched well with the size of the native meniscus. The average anteroposterior (AP) length (mean ± standard deviation) of the native menisci was 49.1 ± 2.7 mm, where the AP dimension of the meniscus prosthesis is 48.9 mm. This results in a mean absolute dimensional difference in AP length of 3.7 ± 2.6%.

Under the highest axial load applied in this study, i.e. 1000 N, total medial meniscectomy increases the peak pressures compared to the intact native meniscus from 2.4 to 5.5 MPa. After implantation of the medial meniscus prostheses, the peak pressure is decreased to 3.7 MPa. The average peak pressure was higher for the meniscus prosthesis than for the native meniscus, but this difference of 1.3 MPa was not statistically significant.

Under an axial load of 500 N, the medial mean pressure in the meniscectomized knee (0.9 MPa) was also higher than with an intact native meniscus (0.4 MPa) and higher than with an implanted meniscus prosthesis (0.5 MPa). The mean pressures increased when increasing the load to 1000 N, to 0.6 MPa for the native meniscus, 1.6 MPa for total meniscectomy and 0.9 MPa for the meniscus prosthesis. For both axial loading conditions, no statistically significant differences were found between the native meniscus and the meniscus prosthesis.

The contact area on the medial tibial plateau was largest with the native meniscus in place, with 543 and 607mm^2^ under 500 and 1000 N, respectively. The contact area decreased after total medial meniscectomy, to 296 and 329mm^2^. A statistically significant increase compared to meniscectomy was not found for the meniscus prosthesis, with contact areas of 335 and 405mm^2^, respectively.

No significant differences were found in the ratio between the loads transferred through the medial native meniscus or meniscus prosthesis and through the direct tibiofemoral contact. Both the native meniscus (from 61 to 51%) and the meniscus prosthesis (from 59 to 43%) seem to transfer a relatively smaller amount of load when the total axial load is increased from 500 to 1000 N, but none of the differences was statistically significant.

**Fig. 10:**
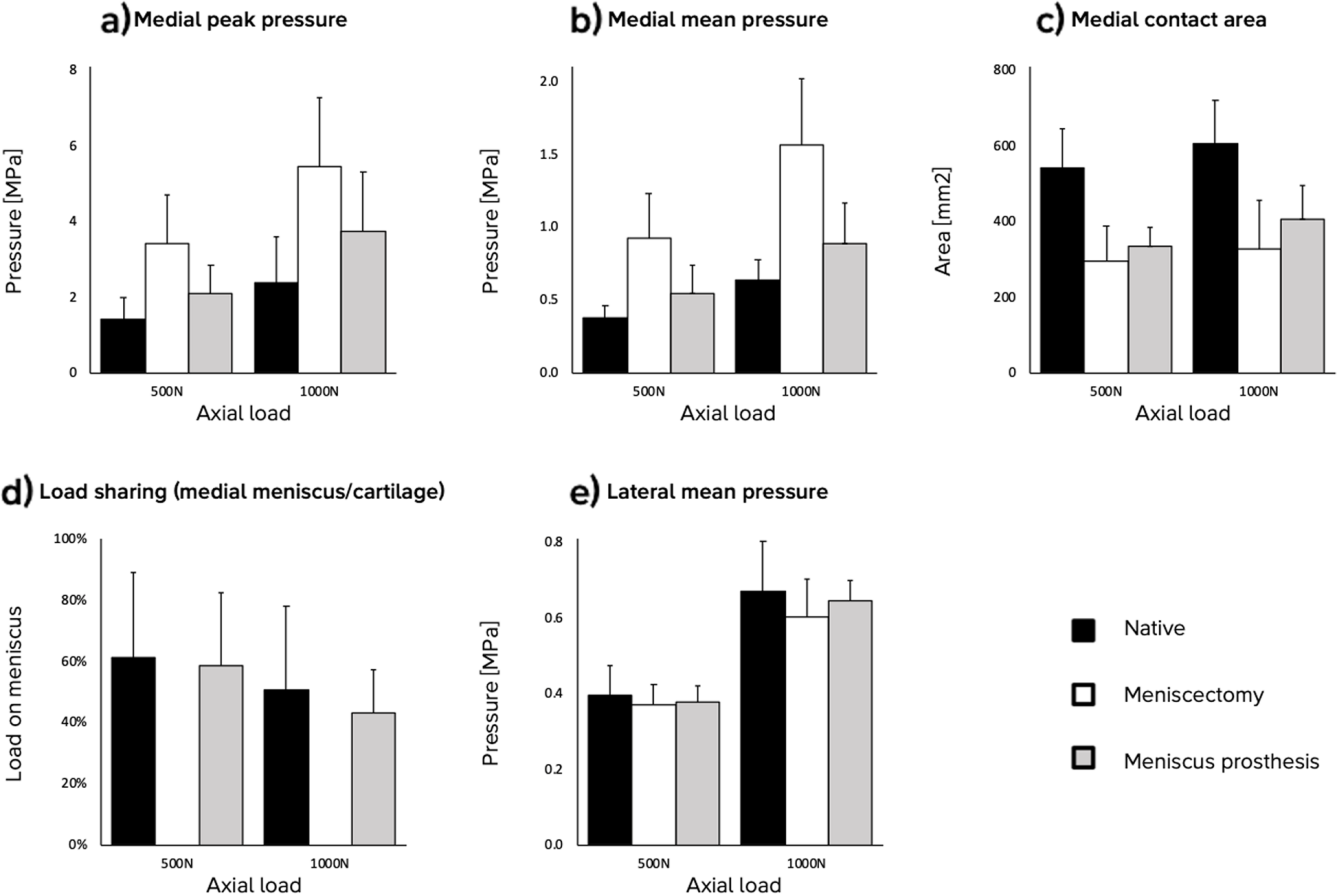
Mean and standard deviation of the biomechanical outcome of the test in cadaveric knee joints, split into medial peak pressure, medial mean pressure, medial contact area, load sharing between the meniscus or prosthesis and the direct tibiofemoral contact, and lateral mean pressure

No significant effect of the meniscal conditions on the mean contact pressures on the lateral tibial plateau was observed. This was the case for both 500 and 1000 N of applied axial load. Detailed results are shown in Fig. 10, Tables 2 and 3.

**Table 2:** Mean and standard deviation of the biomechanical outcomes from the cadaveric study

Force	Condition	Medial peak pressure (MPa)	Medial mean pressure (MPa)	Medial contact area (mm^2^)	Load sharing^a^ (%)	Lateral mean pressure (MPa)
500 N	Native	1.4 ± 0.6	0.4 ± 0.1	543 ± 102	61 ± 28	0.4 ± 0.1
	Meniscectomy	3.4 ± 1.3	0.9 ± 0.3	296 ± 92	-	0.4 ± 0.1
	Prosthesis	2.1 ± 0.7	0.5 ± 0.2	335 ± 51	59 ± 24	0.4 ± 0.0
1000 N	Native	2.4 ± 1.2	0.6 ± 0.2	607 ± 114	51 ± 27	0.7 ± 0.1
	Meniscectomy	5.5 ± 1.8	1.6 ± 0.5	329 ± 123	-	0.6 ± 0.1
	Prosthesis	3.7 ± 1.6	0.9 ± 0.3	405 ± 91	43 ± 14	0.7 ± 0.1

^a^ Percentage of medial compartment load transferred through the meniscus or the prosthesis

**Table 3:** Differences in biomechanical outcomes between the different conditions in the cadaveric study

Outcome	Force	Condition 1	Condition 2	Difference^a^	95% CI	*P*-value
Medial peak pressure (MPa)	500 N	Native	Meniscectomy	2.0	[0.5, 3.5]	< 0.05
		Meniscectomy	Prosthesis	-1.3	[-2.9, 0.2]	NS^b^
		Native	Prosthesis	0.7	[-0.8, 2.2]	NS
	1000 N	Native	Meniscectomy	3.1	[1.5, 4.6]	< 0.001
		Meniscectomy	Prosthesis	-1.7	[-3.3, -0.2]	< 0.05
		Native	Prosthesis	1.3	[-0.2, 2.9]	NS
Medial mean pressure (MPa)	500 N	Native	Meniscectomy	0.6	[0.3, 0.8]	< 0.001
		Meniscectomy	Prosthesis	-0.4	[-0.8, -0.1]	< 0.05
		Native	Prosthesis	0.2	[-0.1, 0.5]	NS
	1000 N	Native	Meniscectomy	0.9	[0.6, 1.2]	< 0.001
		Meniscectomy	Prosthesis	-0.7	[-1.0, -0.4]	< 0.001
		Native	Prosthesis	0.3	[-0.0 0.5]	NS
Medial contact area (mm^2^)	500 N	Native	Meniscectomy	-247	[-361, -133]	< 0.001
		Meniscectomy	Prosthesis	39	[-75, 153]	NS
		Native	Prosthesis	-208	[-322, -94]	< 0.001
	1000 N	Native	Meniscectomy	-278	[-392, -164]	< 0.001
		Meniscectomy	Prosthesis	76	[-38, 190]	NS
		Native	Prosthesis	-202	[-316, -88]	< 0.001
Load sharing^c^ (%)	500 N	Native	Prosthesis	-3	[-30, 25]	NS
	1000 N	Native	Prosthesis	-8	[-35, 20]	NS
Lateral mean pressure (MPa)	500 N	Native	Meniscectomy	-0.0	[-0.1, 0.1]	NS
		Meniscectomy	Prosthesis	0.0	[-0.1, 0.1]	NS
		Native	Prosthesis	-0.0	[-0.1, 0.1]	NS
	1000 N	Native	Meniscectomy	-0.1	[-0.2, 0.0]	NS
		Meniscectomy	Prosthesis	0.0	[-0.0, 0.1]	NS
		Native	Prosthesis	-0.0	[-0.1, 0.1]	NS

^a^ Mean difference between condition 1 and 2: A positive difference means an increase from condition 1 to condition 2

^b^ Not significant, meaning *p* > 0.05

^c^ Percentage of medial compartment load transferred through the meniscus

## Discussion

This study showed that a flexible, anatomically shaped meniscus prosthesis is able to significantly reduce peak and mean pressures in a knee joint after total medial meniscectomy. This improvement is achieved by load sharing between the meniscus prosthesis and the direct tibiofemoral cartilage contact. This load sharing phenomenon did not occur with the stiff first-generation prosthesis, as demonstrated in the test in the artificial knee joint. No significant differences in pressures on the medial tibial plateau between the native meniscus and the second-generation meniscus prosthesis could be demonstrated in this study. Despite the absence of the reinforcing core in the body, the flexible prosthesis was not extruded from the knee joint and therefore sufficiently contributed to the load transfer from the femoral condyle to the tibial plateau during initial use.

The magnitude of peak pressures, mean pressures and contact areas in the native and the meniscectomized knee joint are within the range of contact pressures reported in earlier studies [2, 14, 21, 27, 31]. The main difference with these studies is that the current study was performed in a more physiological environment (i.e. wet and 37 °C), in order to try to mimic the conditions in the knee joint. The importance of testing under these conditions is demonstrated by the test in the artificial knee joint, where the relative amount of load transferred trough the meniscus prosthesis considerably decreased compared to the dry test at room temperature.

**Fig. 11:**
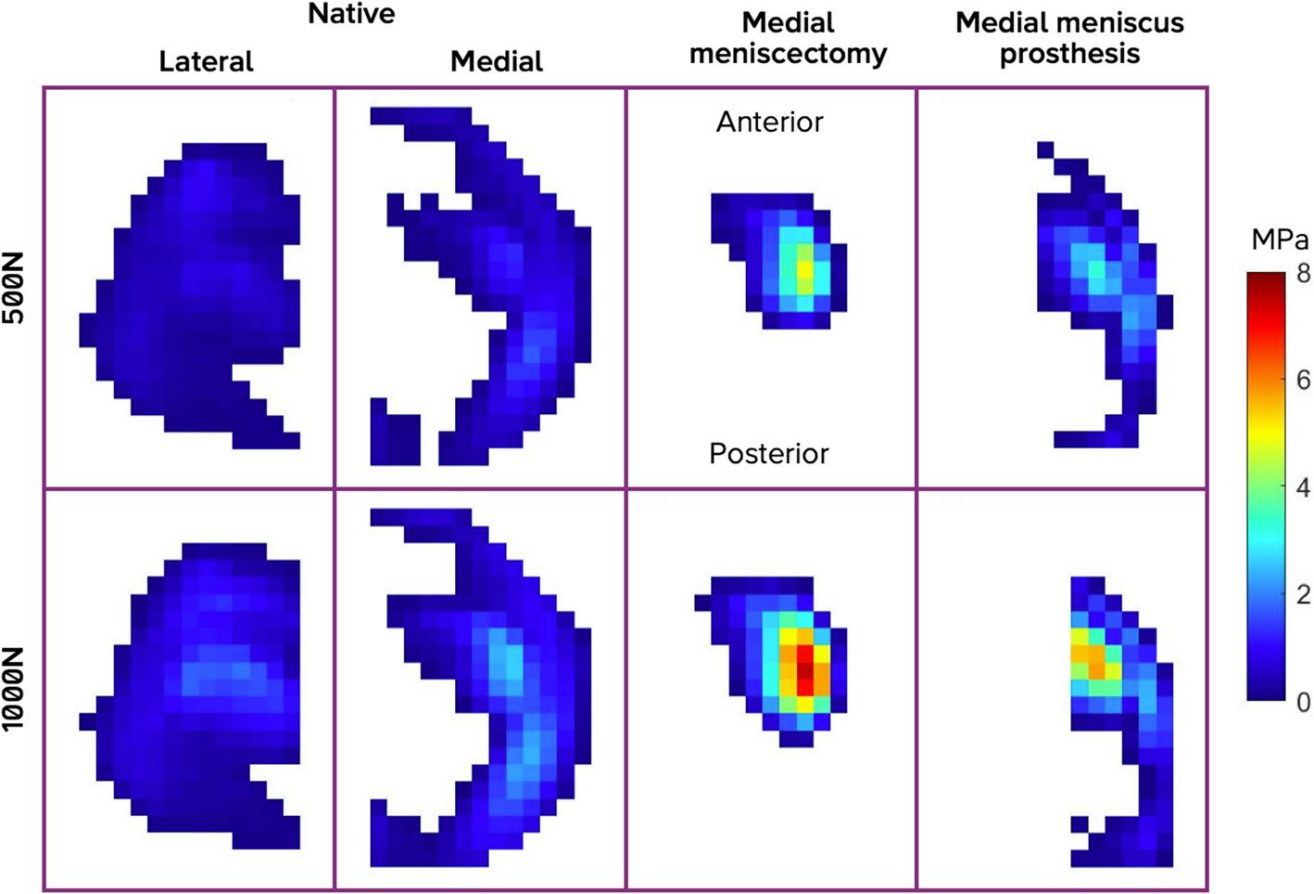
Representative example of the pressure distribution in the different conditions in a cadaveric knee joint

The anterior location of the high-pressure areas (Fig. 11), is consistent with findings during unicompartmental arthroplasties, where typically the tibial cartilage is found to be eroded in the anteromedial area of the tibial plateau [30]. This may be an indication that long-term exposure to high pressures may lead to cartilage degeneration and pain [25]. Although a direct causal relationship between contact pressure and pain has never been demonstrated, meniscal allografts have proven to both decrease tibial contact pressures [11, 27] and relieve pain [23]. An alternative, free-floating medial meniscus prosthesis also demonstrated to decrease tibial contact pressures [22] and reduce pain in patients with partial meniscectomy [33]. Based on the comparison with alternative treatments and the outcomes of this study, the meniscus prosthesis system presented here is expected to relieve pain in patients who have undergone (sub-)total medial meniscectomy. The positive results of the patient with the remaining first-generation prosthesis in the first-in-human clinical investigation corroborate this hypothesis [24]. The outcomes of the clinical investigation suggest that even the stiff version of the meniscus prosthesis may eventually adapt to the geometry of the knee joint through permanent deformation after two years of continued loading. The results indicate that the meniscus prosthesis has the potential to reduce pain in the affected knee compartment, provided that it has a good fit and is positioned correctly [24].

The load sharing ratio between the meniscus or prosthesis and the direct tibiofemoral contact (i.e. the percentage of the medial compartment load that is transferred through the meniscus) found in this study was within the expected range [5, 29]. At different loads, no differences in load sharing capabilities were found between the native meniscus and the second-generation meniscus prosthesis. This finding may indicate a similar behaviour in terms of extrusion, i.e. elastic deformation caused by the axial load on the femur condyle in combination with the wedge-shaped cross-section of the meniscus and the prosthesis. In the cadaveric study, approximately half of the load was carried by the direct tibiofemoral contact, while the results of the test in the artificial knee joint showed that hardly any load sharing occurred with the stiff first generation of the prosthesis.

This study has several limitations, as mimicking the complex interaction between tibia, femur and the deformable meniscus is highly challenging from an experimental point of view. One of the main limitations of the test in the artificial knee joint is the lack of flexibility in the metal femur and the plastic tibia. Although the deformation of the center of the UHMWE tibial plateau simulates the physiological deformation to some extent [8], the rigid material does not adapt to the contacting geometry as the natural cartilage would. The same applies to the metal femoral component. These limitations probably lead to higher pressures in the direct tibiofemoral contact, as a result of a smaller contact area. It is therefore recommended to only use an artificial knee setup, like the one introduced in this study, for direct and qualitative comparison between different test cases. For assessment of *in vivo* mechanical performance and comparison with other meniscal conditions (e.g. meniscectomy), cadaveric studies are preferred.

Ideally, the cadaveric test performed in this study would also have included the previous, stiffer version of the prosthesis. Unfortunately, it was not feasible to subsequently use the screw fixation and the tape fixation methods in the same knee joint, due to the different drill holes required for both techniques. Therefore, it was decided to perform the first part of this study (i.e. the comparison between the flexible and the rigid prosthesis) in an artificial knee joint, which ensures reproducible results.

The cadaveric experiments are also subject to other limitations. Due to the fixation to the tibial plateau (i.e. the presence of anchoring tapes), the pressure-sensitive sensor could not be placed as laterally on the medial plateau as desired, which hindered the measurement of pressures underneath the ends of the prosthesis horns. Therefore, the contact area in the situation with an implanted meniscus prosthesis is probably underestimated in this study. Based on the pressure maps as shown in Fig. 11, one could expect that the meniscus prosthesis does considerably increase the contact area relative to a knee joint that underwent total medial meniscectomy, but not to the level of the native meniscus.

The cadaveric test setup provides a certain amount of freedom in varus/valgus rotation, to allow self-alignment of the joint after intervention. The axis of this varus/valgus rotation is defined by the position and direction of the steel rod (Fig. 8) in the tibia. Due to the design of the test setup and the anatomy of the knee joint the rod was placed in the tibia, a couple of centimetres below the joint line. This impedes the freedom of varus/valgus rotation, which in combination with restricted mediolateral translations limits the self-alignment capabilities of the knee joint.

This may have resulted in a misjudgement of the effect of the different meniscus conditions on the load distribution between the medial and lateral compartment. This limitation could therefore be part of the explanation why no differences in lateral contact mechanics were found in this study. A similar study also found no effect of meniscectomy and implantation of a medial meniscus prosthesis on the lateral knee compartment, but varus/valgus rotation was completely fixed in this study [22].

This study only included axial loading in full extension, while the tibial contact pressures may be higher under different flexion angles [2, 14, 21]. Although the highest physiological loads during gait occur at only 15° of flexion [3, 9], full extension may prevent anteroposterior translation and varus/valgus rotation. Therefore, some differences in biomechanical outcomes may be expected in different flexion angles. In this study, flexion angles are limited by the experimental setup, which does not allow for testing under different flexion angles inside the water bath. Additionally, prolonged testing was not desirable, due to the deterioration of the cadaveric soft tissues in a warm and wet environment, and the potential effect of shear forces in flexed knee joints [31] and prolonged exposure to water [27] on the pressure-sensitive sensors.

Finally, to prevent failure of the cadaveric bones, which occurred during pilot testing, and to prevent overloading of the pressure-sensitive sensors, sub-physiological loads were applied. Higher axial loads would probably have led to higher contact pressures. The main interest, however, was focussing on the differences between the different meniscal situations, and it is not expected that higher loads and larger flexion angles would have a large impact on the relative differences found for all outcome measures of this study.

## Conclusions

The anatomically shaped PCU medial meniscus prosthesis presented in this study was able to significantly improve the contact pressures on the tibial plateau of knee joints that underwent a total medial meniscectomy, by achieving load sharing between the prosthesis and the direct tibiofemoral cartilage contact. Although the biomechanical performance of the native meniscus could not be reproduced completely, the meniscus prosthesis has the potential to relieve pain in patients who have undergone previous (sub-)total medial meniscectomy, which needs to be substantiated in a clinical study.

## Data Availability

The dataset supporting the conclusions of this article will be made available after publication of the article.

## Abbreviations

ACL: Anterior cruciate ligament
AP: Anteroposterior
CI: Confidence interval
CoCr: Cobalt chrome
EtO: Ethylene oxide
MAT: Meniscal allograft transplantation
NS: Not significant
PCU: Polycarbonate urethane
PEEK: Polyether ether ketone
PET: Polyethylene terephthalate
PMMA: Polymethyl methacrylate
RT: Room temperature
Ti-6Al-7Nb: Titanium alloy, containing 6% aluminium and 7% niobium
UHMWPE: Ultra-high molecular weight polyethylene
